# Characterization of the complete mitochondrial genome of the Qiaoke sheep (*Ovis aries*)

**DOI:** 10.1080/23802359.2021.1914519

**Published:** 2021-04-26

**Authors:** Yi zhou Tan, Renqing Dingkao, Dawei Li, Shumeng Yang, Liang Chunnian

**Affiliations:** a Key Laboratory of Yak Breeding Engineering of Gansu Province, Lanzhou Institute of Husbandry and Pharmaceutical Sciences, Chinese Academy of Agricultural Sciences, Lanzhou, China; b Gannan Tibetan Autonomous Prefecture Institute of Animal Husbandry Research, Gansu, China; c Wuwei Agricultural Product Quality and Safety Supervision and Management Station, Gansu, China

**Keywords:** Qiaoke sheep, mitochondrial genome, phylogenetic analysis

## Abstract

Qiaoke sheep (*Ovis aries*) is a local sheep breed in Gansu province, China. It is a kind of Tibetan sheep that used for both meat and wool after long-term breeding. In this paper, the complete mitochondrial genome of Qiaoke sheep was sequenced. The total length of the mitochondrial genome is 16616 bp, and the base composition is 33.65% A, 13.14% G, 25.88% C and 27.33% T. The genome has a total of 37 genes, including 13 protein-coding genes, 22 tRNA genes, two ribosomal RNA genes and a control region (D-loop region). This complete sequence would enlarge useful genomic information for further studies.

It is reported that modern sheep originated from five mtDNA lineages (A–E), but very little or basic information is known about the origins of majority sheep breeds (Rocha et al. 2011). Tibetan sheep play agricultural, economic, cultural, and even religious roles in the Qinghai-Tibetan Plateau areas in China and provide meat, wool, and pelts for the local people (Zhao et al. 2011). Qiaoke sheep have thick long hair braids, large body and good meat performance (Ji et al. 2007). Mitochondria has long been recognized as an important organelle related to disease, apoptosis, aging and metabolism (Boore 1999). The mtDNA is an important tool for assessing the maternal origin, phylogeny and population structure of livestock (Xia et al. 2019). This study is the first one to report the complete mtDNA sequence of Qiaoke sheep and a comparison with other breeds was performed.The unveiling of the mtDNA sequence of Qiaoke sheep will have a significant role to play in the further studies on sheep evolution and domestication. It will enhance germplasm conservation and breeding programs of *O. aries*.

The three blood samples of Qiaoke sheep were collected from Ma-qu County, the southwest of Gannan Tibetan Autonomous Prefecture in Gansu Province, China (100°45′45″∼102°29′00″ E, 33°06′30″∼34°30′15″N). The blood samples were stored in −80 °C refrigerator in the sample storage room of key laboratory of yak breeding engineering of Gansu province. According to the manufacturer's instructions, the EasyPure Blood Genomic DNA Kit (transgen, Beijing, China) was used to extract total genomic DNA from blood samples. The extracted DNA is stored at −20 °C. The mtDNA was amplified with 11 primer pairs in stages by PCR using the primers designed by Guo Jun (Guo 2007). The PCR products were verified by 1% agarose gel electrophoresis. According to the manufacturer's instructions, the EasyPure Quick Gel Extraction Kit (Transgen, Beijing, China) was used for the purification of the tested strips. Then the strips were sent to Qingke Biotechnology Co., Ltd. for sequencing. The sequencing results were assembled using DNAStar Lasergene, ver. 7.1 software. The Metascape was used to annotate the mitochondrial genome.

In this study, the complete mitochondrial genome sequence of Qiaoke sheep (GenBank acc. no.MW200986) were deposited in NCBI. It is 16616 bp circular closed loop structure, including 13 protein-coding genes, 22 transfer RNA genes, 2 ribosomal RNA genes and a control region (D-loop region). In line with other sheep breeds, the arrangement of the genes is a double strand and circular DNA sequence (Yang et al. 2017). It contains the typical structure including two non-coding regions: the origin of light-strand replication (OL) and control region (D-loop region), 2 ribosomal RNA genes (12S and 16S rRNA), 13 protein-coding genes (PCGs), and 22 transfer RNA genes (Mariotti et al. 2013). The overall base composition of the genome is 33.65% A; 27.33% T; 25.88% C and 13.14% G, and the percentage of A + T (60.98%) was found to be higher than G + C (39.02%). The minimal and maximal lengths of overlapped regions are 4 bp and 263 bp. Most of these genes are encoded on the H-strand, except for the ND6 gene and 8 tRNA genes that are encoded on the L-strand. A total of 13 open reading frames of protein-coding sequences have the typical ATN (N refer to A or G) initiation codon. Seven genes used TAA as their termination codon, whereas ND2 and Cyt b terminated with TAG and AGA, respectively. In addition, there are four genes (COX3, ND3, ND4 and ND6) that were found with an incomplete stop codon. Within mtDNA sequences, D-loop regions are highly variable sequences that are usually used in the phylogenetic differentiation in many animal species (Israa and Mohammed Baqur 2020). The control region (CR) or D-loop is 1180 bp in length and is positioned between tRNA-Pro and tRNA-Phe.

In order to determine the phylogenetic position of Qiaoke sheep, MEGA ClustalW, ver. 5.1 software was used to construct a neighbour-joining (NJ) phylogenetic tree that contained the complete mitochondrial genome sequence of 13 sheep breeds including Qiaoke sheep. The results showed that Qiaoke sheep was closely related to Ovis aries breed Awassi (Figure 1). In conclusion, the complete mitochondrial genome sequence of Qiaoke sheep can be further used for phylogenetic reconstruction and conservation strategies of the endemic species.

**Figure 1.**
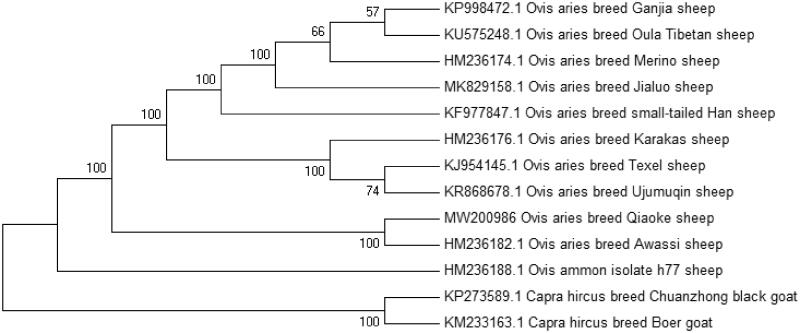
Phylogenetic relationships of mitochondrial genomes of 13 breeds based on the neighbor-joining (NJ) methods. The model is Kimura 2—parameter, the result was validated by 1000 bootstraps and The bootstrap values are shown next to the branches.

## Data Availability

The genome sequence data that support the findings of this study are openly available in GenBank of NCBI at (https://www.ncbi.nlm.nih.gov/) under the accession no. MW200986.
